# Ginkgolic acid inhibits fusion of enveloped viruses

**DOI:** 10.1038/s41598-020-61700-0

**Published:** 2020-03-16

**Authors:** Ronen Borenstein, Barbara A. Hanson, Ruben M. Markosyan, Elisa S. Gallo, Srinivas D. Narasipura, Maimoona Bhutta, Oren Shechter, Nell S. Lurain, Fredric S. Cohen, Lena Al-Harthi, Daniel A. Nicholson

**Affiliations:** 1 Department of Neurological Sciences, Rush University Medical Center, Chicago, IL USA; 2 Department of Microbiology and Molecular Cell Biology, Eastern Virginia Medical School, Norfolk, VA USA; 3 Department of Physiology and Biophysics, Rush University Medical Center, Chicago, IL USA; 4 Independent researcher, Chicago, IL USA; 5 Department of Microbial Pathogens and Immunity, Rush University Medical Center, Chicago, IL USA

**Keywords:** Membrane biophysics, Antivirals, Herpes virus

## Abstract

Ginkgolic acids (GA) are alkylphenol constituents of the leaves and fruits of *Ginkgo biloba*. GA has shown pleiotropic effects *in vitro*, including: antitumor effects through inhibition of lipogenesis; decreased expression of invasion associated proteins through AMPK activation; and potential rescue of amyloid-β (Aβ) induced synaptic impairment. GA was also reported to have activity against *Escherichia coli* and *Staphylococcus aureus*. Several mechanisms for this activity have been suggested including: SUMOylation inhibition; blocking formation of the E1-SUMO intermediate; inhibition of fatty acid synthase; non-specific SIRT inhibition; and activation of protein phosphatase type-2C. Here we report that GA inhibits Herpes simplex virus type 1 (HSV-1) by inhibition of both fusion and viral protein synthesis. Additionally, we report that GA inhibits human cytomegalovirus (HCMV) genome replication and Zika virus (ZIKV) infection of normal human astrocytes (NHA). We show a broad spectrum of fusion inhibition by GA of all three classes of fusion proteins including HIV, Ebola virus (EBOV), influenza A virus (IAV) and Epstein Barr virus (EBV). In addition, we show inhibition of a non-enveloped adenovirus. Our experiments suggest that GA inhibits virion entry by blocking the initial fusion event. Data showing inhibition of HSV-1 and CMV replication, when GA is administered post-infection, suggest a possible secondary mechanism targeting protein and DNA synthesis. Thus, in light of the strong effect of GA on viral infection, even after the infection begins, it may potentially be used to treat acute infections (e.g. Coronavirus, EBOV, ZIKV, IAV and measles), and also topically for the successful treatment of active lesions (e.g. HSV-1, HSV-2 and varicella-zoster virus (VZV)).

## Introduction

Ginkgolic acids are alkylphenol constituents of the leaves and fruits of *Ginkgo biloba. Ginkgo biloba* extracts (GBE) have been used as herbal supplements since at least the 16^th^ century and remain widely in use^1^. Major constituents of GBE include terpine trilactones (ginkgolide A, B, C, J, and bilobalide), flavonoid glycosides (quercetin and rutin), as well as Ginkgolic acids^2^. Ginkgolic acids are a mixture of several 2-hydroxy-6-alkylbenzoic acids in which the most common alkyl chains contain 13, 15, or 17 carbons. The 15 and 17 carbon chains are unsaturated at positions 8 and 10, respectively. The 3 Ginkgolic acid (GA) structures are, therefore, designated C13:0, C15:1, and C17:1 (Table S1)^3^.

GA has shown pleiotropic effects *in vitro*, including: antitumor effects through inhibition of lipogenesis; decreased expression of invasion associated proteins through AMPK activation; potential rescue of amyloid-β (Aβ) induced synaptic impairment; and inhibition of HIV protease activity as well as HIV viral replication^4–7^. GA was also reported to have activity against *Escherichia coli* and *Staphylococcus aureus*^8^. Several ways in which GA works have been suggested including by SUMOylation inhibition activity; blocking formation of the E1-SUMO intermediate^9^; inhibition of fatty acid synthase^10^; non-specific SIRT inhibition^11^; and activation of protein phosphatase type-2C^12^.

Here we report that GA shows antiviral activity against Herpes simplex virus 1 (HSV-1), Human cytomegalovirus (HCMV), and Zika virus (ZIKV) primarily through viral fusion inhibition. In addition, we show inhibition of entry of a replication-defective non-enveloped adenovirus. The antiviral effects were observed below the cytotoxic threshold. We believe that broad spectrum antiviral activity is achieved through the inhibition of viral entry and that this effect could be therapeutically utilized systemically in the context of severe acute viral disease, or topically for the treatment of cutaneous viral lesions. We show a broad spectrum of fusion inhibition by GA of all three classes of fusion proteins^13^ including: pathogenic human enveloped viruses from Class I (ZIKV, HIV, EBOV, and influenza A virus (IAV)), Class II (Venezuelan equine encephalitis virus (VEEV) and Semliki Forest virus (SFV)), and Class III (vesicular stomatitis virus (VSV) and Epstein Barr virus (EBV)). Taken together, our experiments suggest that GA inhibits viral entry by blocking the initial fusion event.

## Results

In reporting the results of our experiments, we apply the term “infection” to measurements of virus cytopathic effect in permissive cells, and the term “replication” to the measurement of genome copies or protein expression.

We chose to use GA C15:1 as the main GA type in our experiments because it is the one most used experimentally to date. However, we also tested GA C13:0 and GA C17:1 in some of our experiments. Unless stated otherwise, GA C15:1 was used.

### GA IC50 vs. EC50

**Figure 1:**
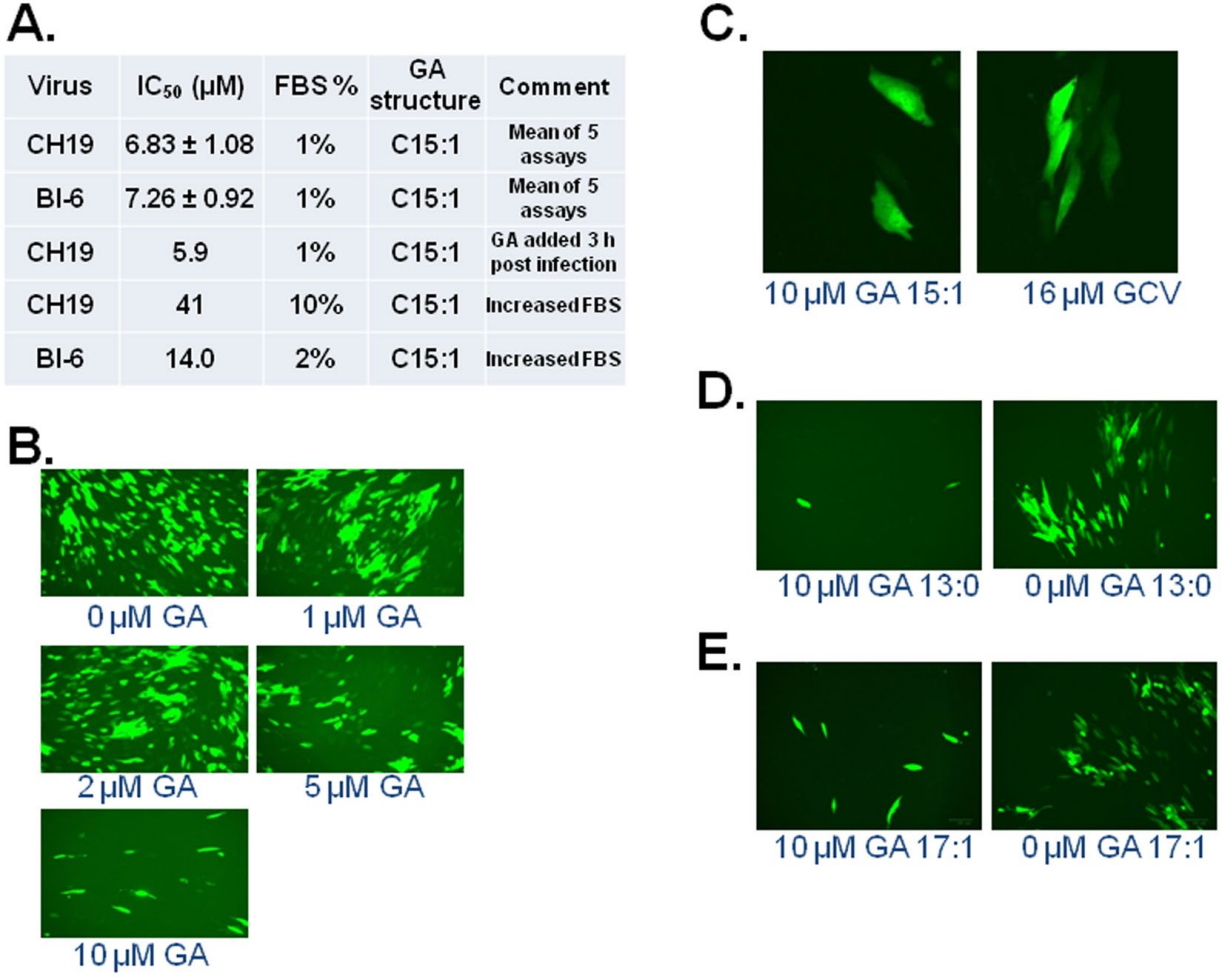
Ginkgolic Acids (C13:0, C15:1, C17:1) inhibit HCMV in a dose-dependent manner and prevents plaque formation. (**A**) Monolayers of human foreskin fibroblasts (HFF) were inoculated with 50–100 cells infected with one of two cell-associated clinical isolates of HCMV (CH19 and BI-6) in a total of 4 wells per drug concentration followed by treatment with medium containing 0–10 µM GA. Viral infection was allowed to progress for 7 days when the mean number of HCMV plaques per concentration was counted, and the IC_50_ was determined. The effects of different concentrations of FBS and time delay on the IC_50_ are also shown. (**B**) Dose-dependent inhibitory effect of GA on infection by the cell-free virus strain PT30CMV-GFP. (**C**) Inhibitory effect of 10 µM GA C15:1 compared to 16 µM GCV on HCMV-GFP cell-free infection. (**D**) Inhibitory effect of 10 µM GA C13:0 on HCMV-GFP infection, and (**E**) Inhibitory effect of 10 µM GA C17:1 on cell-free HCMV-GFP infection. (**B**–**E**) representing average of 20 scanned fields.

The toxicity EC50 of GA on HEp-2 cells in MEM supplemented with 1% fetal bovine serum (FBS) was 27.63 ± 2.21 μM (Fig. S1A) or 63.5 ± 3.2 μM (Fig. S1B) on cells supplemented with 5% FBS. In cells infected with HCMV strain CH19 and supplemented with 1% FBS, the 50% inhibitory concentration (IC50) was 6.83 ± 1.08 μM, and with 10% bovine serum, the IC50 of GA was 41 μM (Fig. 1A). The toxicity EC50 of GA on HFF cells was 14.00 μM ± 0.1 in MEM medium supplemented with 1% FBS (Fig. S1C), and 137.04 μM ± 10.44 (Fig. S1D) in MEM medium supplemented with 10% FBS. The results indicated that the activity and toxicity of GA is affected by the serum concentration in the medium (see discussion).

### GA inhibits HCMV infection in human foreskin fibroblasts (HFF)

To assess the effect of GA on HCMV infection, dilutions of 1 µM to 20 µM were made (Fig. 1A). Monolayers of human foreskin fibroblasts (HFF) were incubated for 1 hour with GA and then infected with two cell-associated clinical isolates of HCMV: CH19^14^ and BI-6^15^. Because neither virus produces extracellular virus, plates for plaque assays were inoculated with 50–100 infected cells per well, and then treated with medium containing 1–20 µM GA, or vehicle control. Viral infection was allowed to progress for 7 days, and then quantitated by plaque reduction assay^14^. GA inhibited infection of BI-6 and CH19 with an IC_50_ of 7.26 ± 0.92 μM and 6.83 ± 1.08 μM, respectively.

To further assess the effect of GA on plaque formation, we used the CMVPT30-GFP strain, which produces cell-free virions. HFF monolayers in 24-well plates pretreated with GA (1–20 µM) for 1 h were inoculated with 50–100 plaque-forming units of CMVPT30-GFP per well.^16^, (Fig. 1B). The number of plaques were counted after 7 days and the IC_50_ was determined. As predicted by the previously-determined IC_50_, at 5 and at 10 µM the antiviral effect was apparent. Furthermore, at 10 µM, only single cells were infected, indicating that there was no cell-to-cell virus transmission. These results may be explained as inhibition of secondary infections or that viral entry may be a target of GA. To verify these results, we compared the inhibition of infection by GA using a plaque reduction assay (PRA) similar to the PRA for ganciclovir (GCV), the preferred antiviral HCMV drug of choice. The results showed that although there was strong inhibition of the virus at 16 µM GCV, there were still visible plaques (>5 adjacent cells showing HCMV cytopathic effect) indicating cell-to-cell transmission of the virions. On the contrary, CMVPT30-GFP infected monolayers that were treated with 10 µM GA only displayed individual infected cells in intact HFF monolayers, (Fig. 1C), thus indicating no cell-cell transmission. We also tested GA C17:1 and GA C13:0. Both GA compounds had a strong inhibitory effect on HCMV infection by PRA, similar to GA C15:1 (Fig. 1D,E). None of the GA structures showed cytotoxicity at their active concentrations, respectively.

### GA inhibits HSV-1 and ZIKV infection

**Figure 2:**
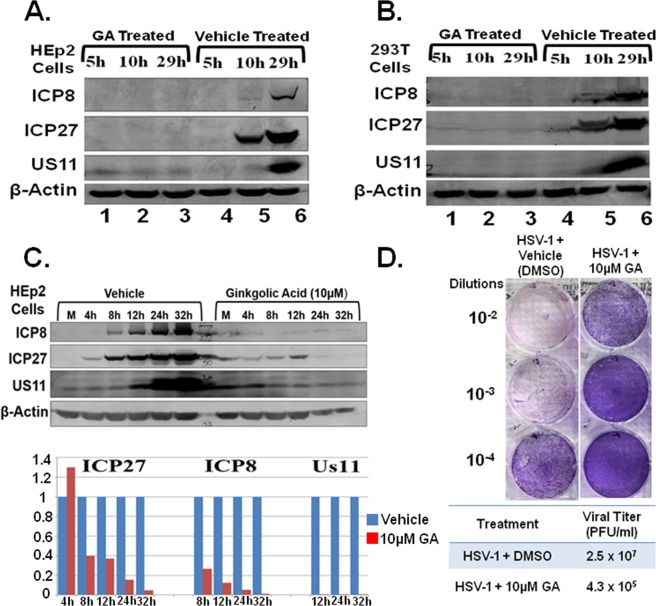
GA inhibits HSV-1 infection. (**A**) 1 × 10^7^ PFU HSV-1 strain F were treated for 1 hour with 50 µM GA (Lanes 1–3) or with vehicle (Lanes 4–6) in serum free DMEM and then were used to infect HEp2 or 293T cells at an MOI of 0.5 in 199V medium with final concentration of 2.5 µM GA. 6-well cultures of HEp-2 (**A**) or 293T (**B**) were then infected, and at 5, 10 and 29 hours post-inoculation, cells were collected and a fraction of the total cell lysate was subject to Western Blot analysis using antibodies directed against ICP8, ICP27, US11 and β-Actin. (**C**) HEp2 cell monolayers were treated with 10 µM GA C15:1 or vehicle for 1 hour in 199V medium and then infected with HSV-1 strain F at 4, 8, 12, 24 and 32 hours post-inoculation cells were collected and a fraction of the total cell lysate was subject to Western Blot analysis using antibodies directed against ICP8, ICP27, US11 and β-Actin. Normalized ratios of protein expression are in the bar graph. (**D**) Titration by plaque assay of Vero cells pretreated with 10 µM GA C15:1 or vehicle and then infected with GFP-HSV-1 strain 17+.

We tested GA on HSV-1, a rapidly replicating and lytic DNA virus, and on ZIKV, an RNA virus which has neither glycoprotein conservation nor common receptors with HCMV or HSV-1. To test whether GA inhibits HSV-1, we designed an experiment to test the direct effect of GA on the virus. For this experiment, we used HEp-2 and 293T cells. 1 × 10^7^ PFU HSV-1 strain F was treated for 1 hour with GA (50 µM) or with vehicle in serum free DMEM and then each of these were used to infect HEp2 or 293T cells at an MOI of 0.5 in 199V medium with a final concentration of 2.5 µM GA. As a control, the virus that was originally treated with the vehicle was then supplemented also with 2.5 µM GA. To test for successful viral infection of HEp2 and 293T cells, production of HSV-1 immediate early (ICP27), early (ICP8), and late (US11) proteins was analyzed by Western Blot (Fig. 2A,B). The results showed that in the treated HSV-1 F stock there was complete inhibition of viral replication, as indicated by lack of protein synthesis. This implies that viral entry may be a target of GA, because direct treatment of HSV-1 with GA blocks downstream HSV-1 protein production. To test the effect of GA on HSV-1 replication, HEp2 cell monolayers were grown to 90% confluency in a 6-well plate, treated with 10 µM GA C15:1 or vehicle for 1 hour in 199V medium, and then inoculated with HSV-1 strain F for protein analysis. HSV-1 ICP27, ICP8, and US11 proteins were detected by Western Blot (Fig. 2C). The results showed profound inhibition of immediate early, early, and late viral proteins. The inhibition of the full temporal range of HSV-1 proteins implies that inhibition of viral replication occurs by blocking the entry of virions into the target cell. To evaluate the effect of GA on progeny virions, the supernatant of GFP-HSV-1 strain 17+ infected Vero cells at an MOI of 1 was collected after 20 h and titered by plaque reduction assay (Fig. 2D). The results showed a significant decrease of approximately 2 log in the GA-treated cell titers.

**Figure 3:**
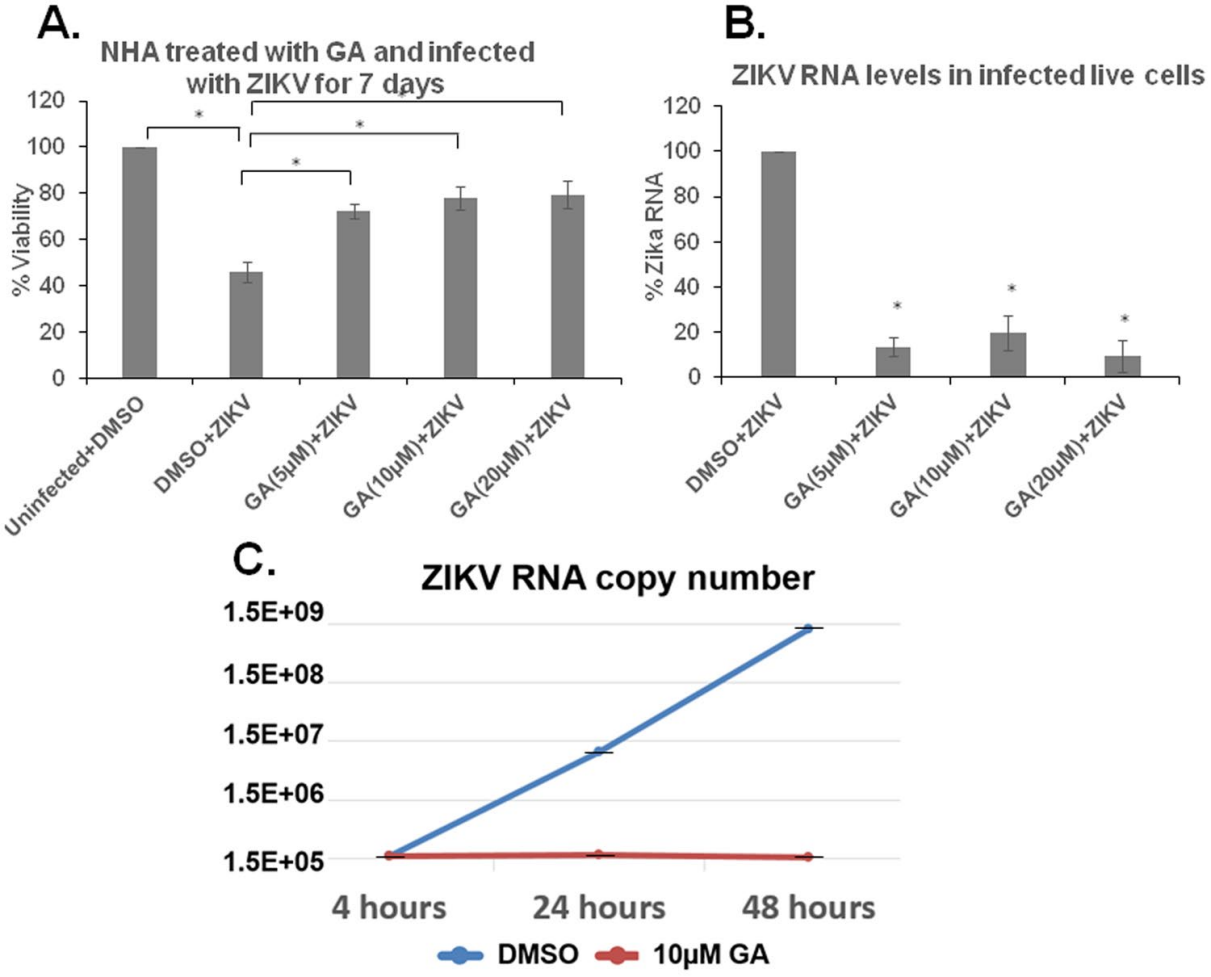
GA inhibits ZIKV infection. (**A**) Viability of NHA infected with ZIKV and treated with GA. NHA were grown in 24-well plates to >80% confluency and treated with GA (0–20 µM) or DMSO for 3 hours and infected with ZIKV strain PRVABC59 at an MOI of 0.3. At 12 hpi, supernatants were carefully removed and replaced with fresh AGM. (**B**) 7 days post infection, samples were analyzed for cell viability by the MTS assay and then live cells were harvested for RNA and processed with Taqman based real-time PCR to quantify ZIKV RNA. Experiments were performed in duplicate three independent times and the data were analyzed by t-test (* indicates p ≤ 0.05). (**C**) Cell free ZIKV in 199V medium was pretreated with 10 µM GA or with DMSO for 1 hour at 37 °C. The pretreated ZIKV was used to infect Vero cells grown in 6 well plates >80% confluency with 0.5 MOI for 1 hour. The infected cells were washed twice with warm DPBS and supplemented with DMEM supplemented with 5% FBS. Supernatant was collected at 4, 24 and 48 hours post-inoculation for cell free viral RNA copy number determination. Experiments were performed in triplicates.

To test the effect of GA on ZIKV infection, NHA were grown to 90% confluency in a 24-well plate, treated with GA or DMSO for 3 hours without serum and then infected with ZIKV strain PRVABC59 at an MOI of 0.3. The next day, supernatant was replaced with fresh medium containing GA or DMSO and cells were incubated at 37 °C, 5% CO_2_. On day 7, viability was determined with the MTS Cell Proliferation Assay (Promega), and then these cells were harvested to extract total RNA for quantification of ZIKV RNA by Taqman based qRT-PCR. The results showed 70% to 80% viability at 5 µM to 20 µM GA, compared to less than 40% viability for the vehicle-treated cells. Furthermore, an 80–90% decrease in ZIKV RNA was observed at 5 µM to 20 µM GA (Fig. 3A,B). We concluded that GA inhibits entry of Zika virions and prevents NHA cell death. This suggests that the mechanism of inhibition that appears to be targeted in HSV and HCMV by GA is conserved among enveloped viruses that have no homologous glycoproteins and use different cell receptors for entry. To test the direct effect of GA on ZIKV, 5 × 10^5^ PFU ZIKV were treated for 1 hour with GA (10 µM/ml) or with vehicle in 199V medium, and then were used to infect Vero cells at an MOI of 0.5. Supernatant was collected at 4, 24 and 48 hours post-inoculation for cell free viral RNA copy number determination (Fig. 3C). The results indicated that the GA-treated ZIKV was completely inhibited, as indicated by cell free ZIKV RNA copy number. This suggests that viral entry of ZIKV, may be a target of GA because direct treatment of ZIKV with GA blocks downstream ZIKV RNA production.

### GA inhibits a non-enveloped adenovirus

**Figure 4:**
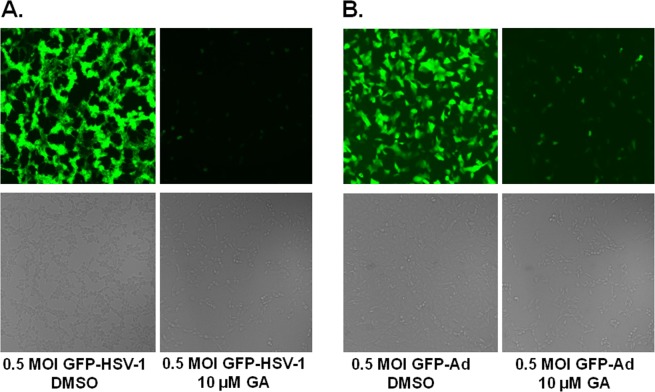
GA inhibits non-enveloped human adenovirus. (**A**) Monolayers of Vero cells were incubated for 1 hour with medium containing 10 µM GA C15:1 or DMSO vehicle. The cells were infected with GFP-HSV-1 virus at an MOI of 0.5 in medium containing 10 µM GA C15:1 for 24 hours. Infection was evaluated by light (lower panels) and fluorescence (upper panels) microscopy. (**B**) Monolayers of Vero cells were incubated for 1 hour with medium containing 10 µM of GA C15:1 or DMSO vehicle. The cells were inoculated with a replication-defective human adenovirus type 5 (dE1/E3) containing GFP (Ad-GFP) at an MOI of 0.5 in a medium containing 10 µM GA C15:1 for 24 hours. Inhibition of entry was evaluated by light (lower panels) and fluorescence (upper panels) microscopy.

To assess the effect of GA on non-enveloped virus, we also tested its antiviral activity on adenovirus, which is internalized by endocytosis. Monolayers of Vero cells were incubated for 1 hour with medium containing 10 µM GA C15:1 or DMSO vehicle. The cells then were inoculated with a replication-defective human adenovirus type 5 (dE1/E3) containing GFP (Ad-GFP) or GFP-HSV-1 virus, at an MOI of 0.5 in a medium containing 10 µM GA C15:1 for 24 hours. The inoculated Vero cells were evaluated by fluorescence microscopy (Fig. 4). The results showed significant HSV-1 inhibition, as well as inhibition of entry of the adenovirus.

### GA inhibits cell fusion induced by all three classes of viral fusion proteins

Our data suggested that GA targets virus-cell fusion. To assess the activity of GA C15:1 on virus-cell fusion we modeled virus-cell fusion by the fusion of cells expressing viral fusion proteins to target cells. Cell-cell fusion was monitored by the spread of fluorescent dyes. Based on crystallographically identified three-dimensional structures, the folds of all virus fusion proteins fall into one of three classes. Effector COS7 cells were transfected to express all three classes of fusion proteins (Table S2), including ZIKV, HIV, EBOV, IAV, SFV, VEEV, VSV and EBV.

**Figure 5:**
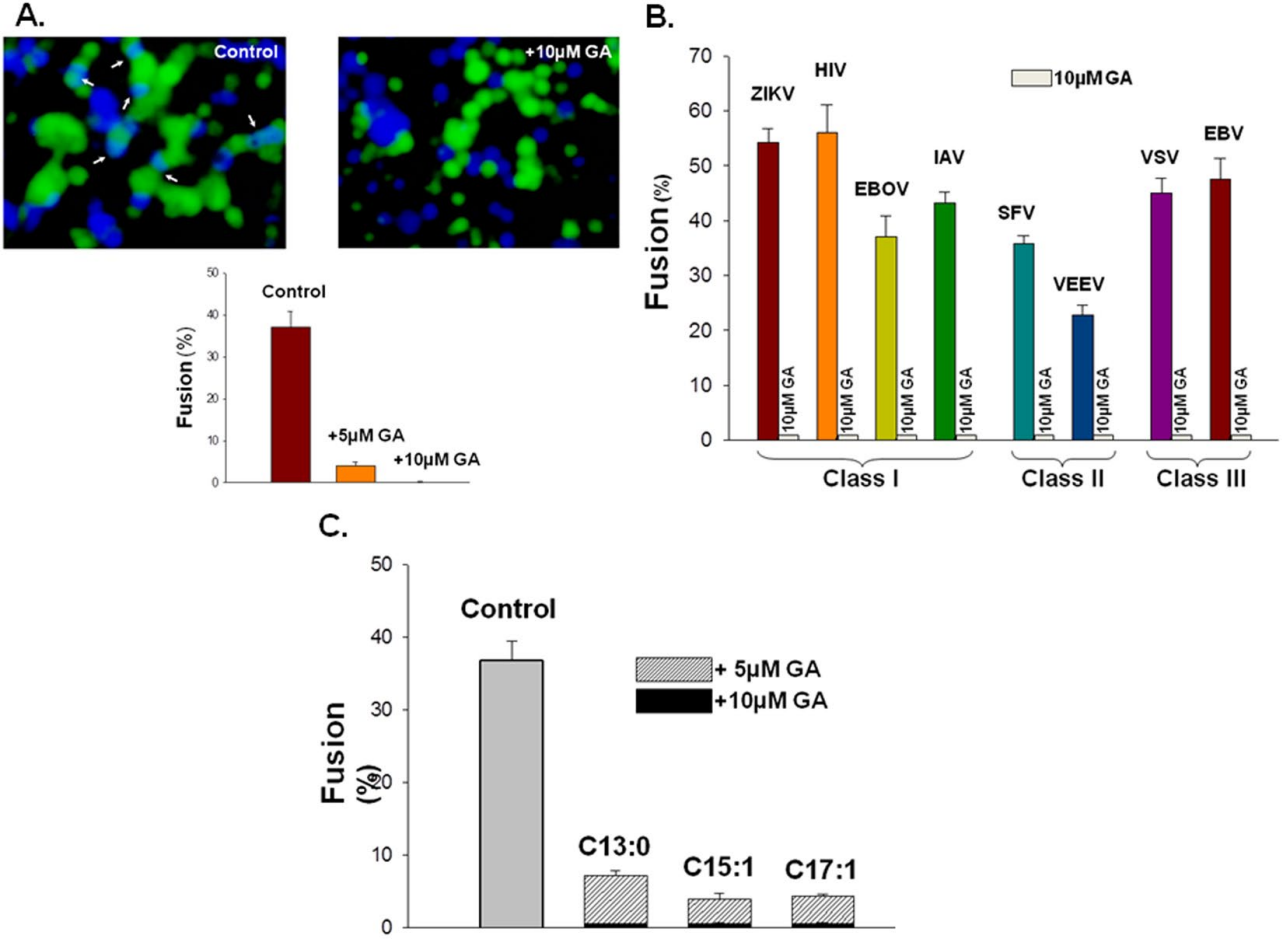
GA inhibits cell fusion induced by all three classes of fusion proteins. (**A**) In the two top panels, typical images used to visualize the extent of fusion in the absence (control, left) and presence of GA (right) are shown. Fused cells (light blue, due to mixing of the green calcein in effector cells and dark blue CMAC in target cells) are marked by arrows in the control. Cells did not fuse in the presence of 10 µM GA. The extent of fusion is quantitated in the bar graph. The experiments used EBOV GP as the fusion protein. (n = 3). For all bars of experiments using cell-cell fusion, means ± SEM are shown. (**B**) Extent of cell-cell fusion induced by fusion proteins from three classes of viral fusion proteins. The proteins for class I are: ZIKV (Zika E), HIV Env, EBOV (Ebola GP), IAV (Influenza HA); for class II: SFV (Semliki forest virus) E1/E2, VEEV (Venezuelan Equine Encephalitis Virus) E; for class III: VSV (Vesicular Stomatitis Virus) G, EBV (Epstein Barr Virus) gB. Effector cells were transfected (except for HIV Env which was stably expressed in a cell line) with the indicated viral proteins. Each was bound and allowed to fuse with target cells. The reduction in fusion caused by adding 10 µM GA is shown as low grey bars labeled 10 µM GA. For every fusion protein, the presence of GA abolished fusion. n = 3 for each fusion protein. (**C**) Effect of two different concentrations of GA with different side chains on cell-cell fusion.

The COS7 cells were loaded with calcein AM and bound to 293T target cells that were either unlabeled or, for purposes of microscopic identification, loaded with the aqueous dye CMAC, as illustrated for EBOV GP-induced fusion (Fig. 5A). We tested the effect of adding GA on cell-cell fusion mediated by four class I, two class II, and two class III fusion proteins. For all eight proteins, 10 µM GA C15:1 completely blocked fusion (Fig. 5B). Furthermore, when EBOV GP was treated with 5 µM or with 10 µM GA C13:0, GA C15:1, or GA C17:1 (Fig. 5C), 10 µM of all GA derivatives completely blocked fusion.

**Figure 6:**
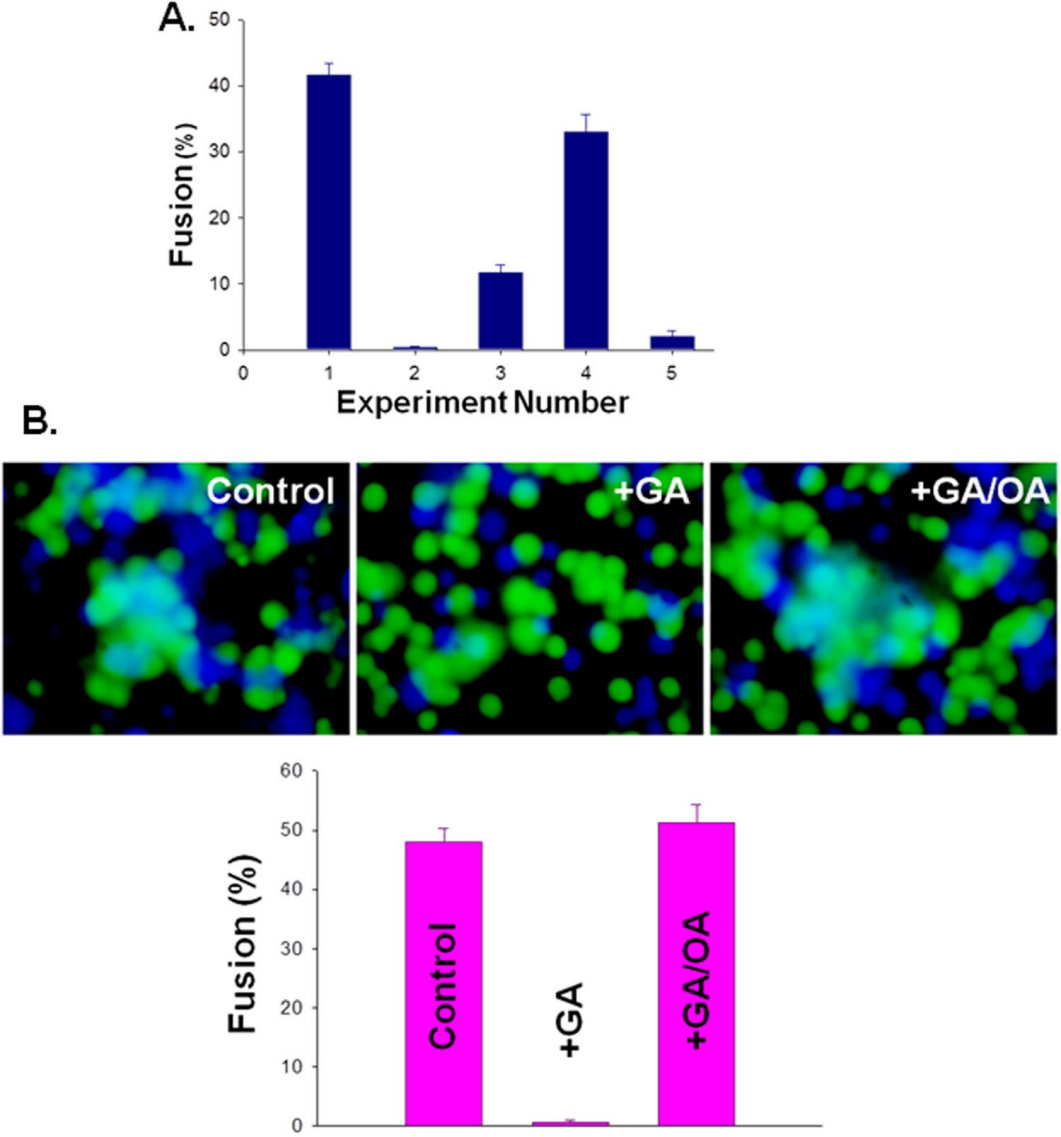
OA together with GA abolished the inhibitory effect of GA. (**A**) Dissecting the stages of fusion affected by the presence of GA. Bar 1, control: GA was not present. Bar 2: GA was maintained throughout the experiments. Bar 3: Effector and target cells were incubated for 30 min. in the presence of 10 µM GA. The neutral pH bathing solution was then replaced by a pH 5.7 solution, which did not contain GA, and this was maintained for 10 min. The low pH bathing solution was replaced by one at neutral pH, also without GA, and fusion was measured. Bar 4: The same protocol was followed as for experiments of bar 3, but prior to acidification, the GA-containing neutral pH solution was replaced by a GA-free neutral pH solution that was maintained for 10 min to allow GA in the membranes to dissociate into the aqueous solution. This almost restored the extent of fusion to that of control. Bar 5: The same protocol as for experiments of bar 3 was used, but GA was maintained through acidification. The subsequent neutral pH wash solution did not contain GA. EBOV GP was the fusion protein for these experiments. (n = 3). (**B**) The inhibition of cell-cell fusion by GA is reversed by the presence of oleic acid (OA). Upper panels: Massive dye mixing was observed for control (left image), but none was observed in the presence of 10 µM GA (middle image). Addition of 250 µM OA along with GA (right image), resulted in the same degree of fusion as for the control. Extent of fusion is quantified in the bar graphs. (n = 3).

When GA was removed, fusion was restored (Fig. 6A, Bar 4), indicating that GA interferes with fusion in a reversible manner. Viral fusion, regardless of fusion protein, proceeds through the creation of hemifusion, an intermediate state in which proximal lipid monolayer leaflets of membranes, in contact with one another, have merged, but the distal monolayers remain distinct. Because inhibition of fusion was universal, independent of viral protein, it is extremely unlikely that GA targeted the fusion proteins themselves. This is consistent with GA inhibiting the creation of the hemifusion intermediate, and universal inhibition would be expected. It is well known that agents conferring positive spontaneous curvature, such as lysophosphatidylcholine (LPC), inhibit fusion induced by viral and non-viral fusion proteins^17^. Likewise, the cone-shaped lipid oleic acid (OA) has a negative spontaneous membrane curvature, which favors hemifusion when present in the outer bilayer, and its presence relieves the inhibition of fusion^18^. The addition of OA together with GA abolished the inhibitory effect of GA (Fig. 6B), indicating that GA induces positive membrane curvature.

### GA inhibition of enveloped viruses correlates with inhibition of viral DNA and protein synthesis

**Figure 7:**
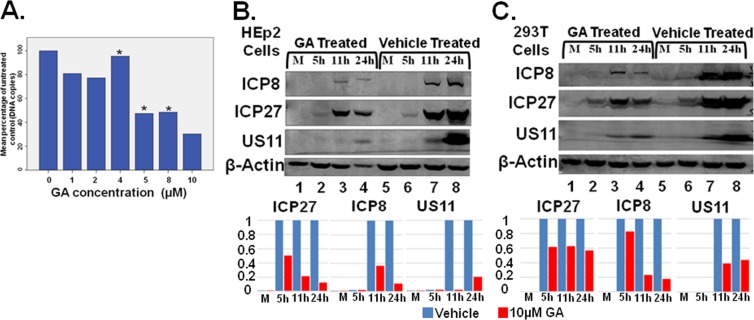
GA inhibits HSV-1 replication by inhibition of viral protein synthesis and HCMV by DNA synthesis. (**A**) Effect of GA on HCMV DNA synthesis. Viral replication is inhibited in the presence of increasing concentration of GA, as determined by decreasing viral DNA copies. DNA was extracted from 4 pooled wells of HFF at 7 dpi, then analyzed by qPCR targeting the viral polymerase gene. DNA copies were reduced in a dose dependent manner by the addition of GA to culture medium as determined by ANOVA analysis with pairwise post-hoc t-test analysis in SPSS. Data are presented as mean percent of control. (*5 µM p = 0.014, 8 µM p = 0.003, 10 µM p = 0.002). 6 well cultures of HEp-2 (B) or 293T (**C**) were infected with HSV-1 strain F at an MOI of 1 for 2 hours, then washed with 199V and supplemented with 10 µM GA (Lanes 1–4) or vehicle (Lanes 5–8). Mock (M), 5, 11 and 24 hours after inoculation, cells were collected and a fraction of the total cell lysate was subject to Western Blot analysis using antibodies directed against ICP8, ICP27, β-Actin and US11. Normalized ratios of protein expression are in the bar graphs.

To validate the PRA observations, we tested whether there is an effect on downstream viral DNA synthesis in HCMV infected cells when treated with GA. HFF were inoculated with HCMV for 3 hours, then washed and incubated with different concentrations of GA or vehicle. DNA was extracted from 4 pooled wells of HFF at 7 days post inoculation, and then analyzed by qPCR targeting the viral polymerase gene. DNA copies were reduced in a dose dependent manner by the addition of GA to culture medium (Fig. 7A). Although HCMV DNA copy numbers were reduced by GA, this could be secondary to inhibition of virus entry rather than direct inhibition of DNA replication.

Viral protein synthesis was determined in HSV-infected cells following GA treatment. Untreated HEp-2 and 293T cells were infected with HSV-1 F’ at an MOI of 1 for 2 hours, allowing the virions to internalize into the cells, then washed with 199V medium and supplemented with 10 µM GA or vehicle. The viral replication was evaluated by Western Blot detection of HSV-1 proteins. Although the infection of HEp-2 and 293T cells had been initiated, the addition of 10 µM GA to the infected HEp-2 and 293T cells inhibited the virus replication from the point of exposure to GA (Fig. 7B,C). The observed effect could be the result of inhibition of secondary infections. However, the early down regulation of HSV proteins, occurring prior to a complete viral replication cycle, suggests that there could be a secondary inhibitory mechanism targeting viral protein synthesis.

## Discussion

The Ginkgolic acids C13:0, C15:1 and C17:1 are commercially available compounds of Ginkgo leaves. The antioxidative activity of GA has been reported to have multiple therapeutic effects including the treatment of cardiovascular disease, HIV infection, bacterial infections such as *Escherichia coli* and *Staphylococcus aureus*, and some tumors^7,8,19,20^. It has been suggested that GA may operate by several other pathways including: inhibition of fatty acid synthase^10^; non-specific SIRT inhibition^11^; activation of protein phosphatase type-2C^12^; suppression of STAT3 activation through induction of PTEN and SHP-1 Tyrosine Phosphatase^21^, and protection against Aβ-induced synaptic dysfunction in the hippocampus^6^.

In this study, we are the first to report the fusion inhibitory effect of GA on enveloped viruses representing the three classes of fusion proteins and the inhibition of a non-enveloped human adenovirus. We also report a potential secondary mechanism of action involving viral DNA and protein synthesis. Our results are consistent with previous reports of the inhibitory effect of GA on DNA and protein synthesis^22^. The mechanism of action through which GA affects DNA and protein synthesis is not yet understood. It may bind to the host cell receptors and activate different cell signaling pathways and/or cause cell cycle arrest, which may explain the inhibitory effect of GA on rapidly dividing cancer cells. It may also enter the cells and work directly on DNA and protein synthesis. Experiments to address these questions are ongoing.

To assess the effect of GA on infectious viruses, we performed GA dose-response experiments. The cells were treated with different GA concentrations ranging between 1 µM and 20 µM. We demonstrated a dose dependent effect of GA on HCMV, HSV-1, and ZIKV. The effect of GA was tested in several cell types including HEp-2 (human epithelial carcinoma), 293T (human embryonic kidney), HFF and NHA (normal human astrocytes). GA was shown to have a viral inhibitory effect in all of the tested cells with no cytotoxicity within the active inhibitory range.

Berg *et al*. evaluated the cytotoxicity and mutagenicity of GA in male Chinese hamster lung fibroblasts (V79 cells)^23^. Their results showed toxicity on cells grown in DMEM supplemented with 10% FBS after 24 hours in GA concentrations above 50 µM, with no mutagenic effect. Ahlemeyer *et al*. reported that 500 µM GA induced neuronal death and activated protein phosphatase type-2C in chick embryonic neurons growing in DMEM with 20% FBS^12^. B.M Hausen, evaluated the sensitizing capacity of GA in guinea pigs, determining 1000 ppm (2.886 mM) GA as safe to avoid inducing an allergic reaction^24^. Viral infections of permissive cells are regularly performed in 199 or MEM medium supplemented with 1% or 2% FBS. However, when we tested the activity and toxicity of GA on the cells used in our research, we also incubated the cells with GA in the cells’ recommended growth medium (see results and Fig. S1). The results indicated that the activity and toxicity of GA is affected by the serum concentration in the medium. We concluded that GA interacts with serum factors, which lowers its antiviral activity, and researchers using GA should address this issue in future experiments. *In vivo* experiments in an animal model are needed to assess the actual therapeutic antiviral effect and cytotoxicity of GA.

GA’s universal inhibition of viral protein-mediated cell-cell fusion indicates that its inhibitory effect is by a common fusion mechanism. LPC also universally blocks membrane fusion; it does so by conferring spontaneous positive curvature, which prevents hemifusion. This block can be relieved, regardless of fusion protein, by the addition of the negative spontaneous curvature agent OA^25,26^. The finding that OA relieves the GA-induced inhibition of EBOV GP-mediated fusion implies that, similar to LPC, GA acts by producing positive spontaneous curvature and this prevents hemifusion. A number of rigid amphipathic fusion inhibitors (RAFI) with positive spontaneous curvature have been shown to inhibit fusion induced by unrelated viral fusion proteins^27^. In the future, it would be interesting to measure the values of the spontaneous curvatures of GA and RAFI and to relate them to the concentration of OA necessary to relieve fusion inhibition. For the inhibition of non-enveloped adenovirus, we suggest that since GA affects lipid bilayer curvature, it would be predicted to affect the endocytic entry of a non-enveloped virus such as adenovirus. In addition, as we report here, GA appears to have potential secondary mechanisms of viral DNA and protein synthesis inhibition, and these would be predicted to be targeted in both enveloped and non-enveloped viruses.

In conclusion, we have shown a consistent inhibitory effect of GA on the fusion of a variety of enveloped viruses, including important pathogens such as EBOV, HIV, ZIKA, HSV-1, HCMV, EBV and IAV. We also have shown inhibition of a non-enveloped human adenovirus, which suggests a potential inhibitory effect on other non-enveloped viruses. Furthermore, we found that GA might possibly inhibit HCMV viral DNA and HSV-1 protein synthesis by a secondary mechanism.

Thus, in light of the antiviral effect of GA on established viral infections of permissive cells, GA potentially could be used to treat acute viral infections (e.g. Coronavirus (COVID-19), EBOV, ZIKV, IAV and measles), and it might be determined to be useful in topical application for the successful treatment of active lesions (e.g. HSV-1, HSV-2 and VZV). Finally, our approach for GA usage to inhibit enveloped virus infection is fundamentally different from traditional microbicidal strategies that target virus genome replication. We anticipate that it could complement other direct antiviral agents and offer a new class of inhibitors of enveloped and non-enveloped viruses.

## Methods

### Cells, viruses and GA

HEp2 cells, obtained from the American Type Culture Collection (ATCC Rockville, MD), were grown in Dulbecco’s Modified Eagle Medium (DMEM) supplemented with 5% fetal bovine serum (FBS). HEK293T/17 (293T) cells obtained from the ATCC were grown in DMEM supplemented with 10% FBS. HSV-1 strain F, a limited-passage isolate, is the prototype strain used in this laboratory^28^. GFP-HSV-1 (strain 17), was a generous gift from Dr. Peter O’Hare^29^. Vero cells obtained from the ATCC were cultured in complete DMEM (cDMEM) containing 10% FBS, 2 mM L-glutamine, 100 U/mL penicillin/streptomycin, and 10 mM Hepes buffer at 37 °C with 5% CO_2_. Fetal-derived Normal Human Astrocytes (NHA, Lonza) were maintained in Astrocyte Growth Medium (AGM, Lonza) supplemented with 0.3% FBS, 30 µl/mL ascorbic acid, 1 µl/mL rhEGF, 30 µg/mL gentamicin, 15 μg/mL amphotericin B, 2.5 µl/mL insulin, and 10 µl/mL L-glutamine. Early passages (1–4) were used in these experiments.

HCMV studies were performed using cell-associated low-passage clinical isolates CH19^14^ and BI-6^15^. CH19 is resistant to the first-line HCMV antiviral drug, ganciclovir (GCV), while BI-6 is susceptible to GCV. Additional experiments were performed using CMVPT30-GFP^16^, which expresses green fluorescent protein and produces extracellular virus.

ZIKV strain PRVABC59 obtained from the American Type Culture Collection (ATCC; Manassas, VA) was propagated in Vero cells grown in T-150 flasks by infection at 1:50 dilution of viral stock (MOI 0.25) in the absence of FBS. The medium was removed and replaced 6 h post-infection (hpi) with fresh cDMEM. Supernatant was collected at 72 hpi, clarified by centrifugation at 350 × g for 5 min, and filtered through a 0.45-μm surfactant-free cellulose acetate membrane. For mock infections, supernatant was collected from uninfected Vero cells and prepared by the same protocol used to make virus stocks. Virus was titered by focus assay. Briefly, infected Vero cells, 24 hpi were fixed and permeabilized using Cytofix/Cytoperm Solution Kit (BD Biosciences) according to the manufacturer’s instructions and stained with a mouse monoclonal antibody (mAb) specific for flavivirus group envelope proteins (1:250; EMD Millipore; clone D1–4G2-4-15) followed by incubation with an anti-mouse IgG - PE (1:1000 dilution). Samples were run through an LSR II flow cytometer and data were collected with FACSDiva software (BD Biosciences) and analyzed using FlowJo Software (TreeStar). Human Adenovirus Type5 (dE1/E3), Cat. No.: 1060, was purchased from Vector Biolabs.

GA C13:0, C15:1, and C17:1, were purchased from Sigma Aldrich, St. Louis, MO. GA was diluted in methanol or DMSO to a concentration of 50 mM. Effects of GA in all assays and models used in this study have been compared to vehicle-treated (i.e. methanol or DMSO) controls. Cells or viral stocks were treated with the indicated concentration of GA.

### Western blot analyses

For HSV-1, cells were harvested at the indicated times, collected by low-speed centrifugation and rinsed in PBS, then resuspended in RIPA lysis buffer and protease inhibitors (Complete Protease Inhibitor; Roche). Approximately 60 μg of protein per sample was subjected to further analysis. Proteins were electrophoretically separated on 8 to 10% denaturing polyacrylamide gels, electrically transferred to nitrocellulose sheets, blocked by 5% BSA in Tween PBS and reacted with the primary antibodies. The mouse monoclonal antibody to US11 (Goodwin Institute for Cancer Research), and the mouse monoclonal antibody to ICP8, were used at a dilution of 1:1,000. The mouse monoclonal antibody to ICP27 was used at a dilution of 1:250. The mouse monoclonal antibody to β-actin (Sigma) was used at a 1:5,000 dilution. Membranes were then reacted with the appropriate secondary antibody conjugated either to alkaline phosphatase or to horseradish peroxidase. Protein bands were visualized with 5-bromo-4-chloro-3-indolylphosphate–nitroblue tetrazolium (Denville Scientific, Inc.) or with ECL Western Blot detection reagents (Amersham Biosciences) according to the manufacturer’s instruction.

### Cytotoxicity tests

Cells grown in 96 or 48 well plates were exposed to different concentrations of GA for 24 h. Cell viability was determined by Alamarblue® assay (Thermo Fisher Scientific, Catalog number: DAL1025). Pair-wise Student t-tests were used to compare the outcome of a treatment as compared to the control. Error bars are SEM (n ≥ 3).

### Viral stock preparation

Confluent Vero cells in 75-cm2 flasks were infected with HSV in 3 ml of 199V (Gibco; 199 medium supplemented with 1% heat-inactivated FBS and 1% penicillin/streptomycin) medium at a multiplicity of infection (MOI) of 0.01 pfu/cell and incubated by gentle shaking for 2 hours at 37 °C with 5% CO_2_ to allow cells to absorb the virus. After 2 hours, 199 V was aspirated from the cell culture and Vero cells were gently washed with DPBS. 3 ml of fresh DMEM/5% FBS medium was added and the cells were incubated for 2 to 3 days until 100% of the cells display cytopathic effect (CPE). When 100% CPE was observed, the flask was placed at −80 °C for 15 min, then allowed to warm up at room temperature and 3 ml of cold sterile milk was added. The cells were collected into a 15 ml tube and kept on ice. To free virus particles from the cellular debris, Vero cells were sonicated three times (letting the cell suspension cool on ice for 1 min between sonications), for 30 seconds using a Branson Sonifier with microtip at an output setting of 4 (approximate Power Output of 15%). The virus stock was aliquoted and transferred to sterile, screw-capped cryovials and was stored at −80 °C. The viral titer was determined by plaque assay.

### HSV-1 plaque assay

GA (10 µM) was incubated on the monolayers of Vero cells (6-well plate) with 199V (Gibco; supplemented with 1% heat-inactivated fetal bovine serum and 1% penicillin/streptomycin) at 37 °C for 1 hour prior to inoculation. Following incubation, the supernatants were aspirated and cells were washed two times with DPBS. GFP-HSV-1 strain 17+^29^, at an MOI of 1 was incubated on the cells with 199V at 37 °C for 24 hours. The supernatant was collected and titered. Various 10-fold dilutions of collected supernatants were incubated on monolayers of fresh Vero cells (6-well plate) at 37 °C on a shaker for 2 h to allow the virus to attach and enter the cells. The supernatants were aspirated and cells were incubated with 20 mg/mL of human serum (Sigma-H4522) in DMEM (Dulbecco’s Modified Eagle’s medium, supplemented with 5% FBS and 1% penicillin/streptomycin) for 3 days at 37 °C in 5% CO_2_ to allow plaque formation. For plaque counting, the cells were fixed with 100% methanol for 5 min. at room temperature and stained with 10% KaryoMax Giemsa stain in distilled water for 20 min. at room temperature. Viral titer was calculated through plaque counting and expressed as plaque-forming units (PFU)/ml.

### ZIKV studies

Cell free ZIKV in 199V medium was pretreated with 10 µM/ml GA or with DMSO for 1 hour at 37 °C. The pretreated ZIKV was used to infect Vero cells grown in 6 well plates >80% confluency with 0.5 MOI for 1 hour. The infected cells were washed twice with warm DPBS and supplemented with DMEM supplemented with 5% FBS. Supernatant was collected at 4, 24 and 48 hours post-inoculation for cell-free viral RNA copy number determination. NHA were grown in 24 well plates to >80% confluency, treated with GA C15:1 (0–20 µM) for 3 h, and then infected with ZIKV at an MOI of 0.3. Supernatant was carefully removed and replaced at 12 hpi with fresh AGM replenished with GA at 0–20 µM concentrations. Cell viability was assessed at 7 days post-inoculation by Celltiter Aqueous One solution cell proliferation assay (Promega, Madison, WI), and live cells were carefully harvested to extract RNA by the RNeasy kit (Qiagen, Germantown, MD). RNA was quantified using a Nanodrop2000 (ThermoFisher), treated with DNaseI (Sigma-Aldrich) for 15 min at room temperature to remove DNA contamination, and subsequently, DNaseI was inactivated by heating at 70 °C for 15 min. cDNA was synthesized from 0.2–1 μg of RNA using Qscript supermix (Quanta Biosciences). Quantitative real-time PCR (qRT-PCR) reactions were performed in a 20 μl solution containing 10 μl TaqMan Gene Expression Master Mix (Life Technologies), 500 nM primers, and 300 nM probe. Reactions were performed in an Applied Biosystems 7900HT sequence detection system (Thermo Fisher Scientific) using SDS2.3 software. The reaction conditions were, 50 °C for 2 min, 95 °C for 10 min, followed by 45 cycles of 95 °C for 15 s and 60 °C for 1 min. Samples were run in duplicate or triplicates, and template controls were included wherever necessary. Fold change in RNA expression was calculated by relative quantification using the comparative C_T_ method with GAPDH as endogenous control. The primers and probe were designed using the PrimerQuest tool (Integrated DNA Technologies). The sequences of the primers were as follows:

ZIKV-F-CGCTGCCCAACACAAGGT; ZIKV-R-, GCTCCCTTTGCCAAAAAGTCCACA, and ZIKV-probe, 5′/56-FAM/ACCTTGACA/ZEN/AGCAGTCAGACACTCAA/3IABkFQ; and human specific GAPDH-F-GGTGTGAACCATGAGAAGTATGA; GAPDH-R-GAGTCCTTCCACGATACCAAAG; and GAPDH-probe, 5′/56-FAM/AGATCATCA/ZEN/GCAATGCCTCCTGCA/3IABkFQ.

### HCMV studies

HFF were grown in 24 well plates to 100% confluency in MEM with 10% FBS to establish viral inhibition of two low-passage clinical strains (BI-6 and CH19) via plaque assay. Cells infected with low-passage HCMV clinical strains (CH19 and BI6) do not release extracellular virus, but they display typical HCMV cytopathic effect (CPE). The percentage of infected cells was estimated visually in HFF monolayers. Dilutions of trypsinized cells were used as inocula for HFF monolayers in 24 well plates. After 7 days in culture, plaques were counted to determine the average number of plaques in 4-well replicates of each dilution. The optimal dilution for each virus strain produced an average of 60–80 plaques per well. CMVPT30-GFP having undergone multiple passages produces extracellular virus. Viral infection was quantitatively determined by PRA of culture supernatant medium. Cells were either exposed to GA for three hours prior to inoculation, or were inoculated prior to addition of medium containing GA at concentrations of 1, 2, 5, 10, and 20 µM in MEM with 1% FBS. One set of four wells was treated with DMSO as a vehicle control. For each experiment the remaining concentrations were represented in quadruplicate wells. Plates were read beginning at 7 days post inoculation (dpi) to determine the GA concentration producing a reduction of 50% in the number of plaques relative to vehicle control wells (IC_50_). Data were normalized to the vehicle controls, and probit analysis in SPSS was used to obtain 95% confidence intervals of the mean IC_50_ values.

### HCMV DNA extraction and quantitation

HCMV infected monolayers were trypsinized and pooled at the termination of the PRA. DNA was extracted from the pooled monolayers using the Blood gDNA kit (Promega, Madison, WI). HCMV DNA was quantitated using a validated in-house assay, which targets the DNA polymerase^30^. Reactions were performed on an Applied Biosystems 7500 (Thermo Fisher Scientific).

### Infection of GFP-expressing viruses

Monolayers of Vero cells were incubated for 1 hour with medium containing 10 µM GA C15:1 or DMSO vehicle. The cells then were inoculated with a replication-defective human adenovirus type 5 (dE1/E3) containing GFP (Ad-GFP) or GFP-HSV-1 virus, at an MOI of 0.5 in a medium containing 10 µM GA C15:1 for 24 hours. Infection was evaluated by fluorescence microscopy.

### Cell-cell fusion vectors

In these experiments, we used cell-cell fusion systems of ZIKV, HIV, EBOV, IVA, VEEV, VSV, SFV and EBV. The plasmids, fusion proteins, and the related references are described in Table S2.

### Fusion experiments

Nucleated cell-cell fusion, experiments were performed as described^31,32^. Calcein-AM was loaded into effector cells expressing the fusion protein, and 7-amino-4-chloromethylcoumarin (CMAC) was loaded into target cells. Mixing of dyes was scored as fusion. Fusion experiments for each viral protein were performed as previously described: EBOV GP^32^; HIV^33^; SFV E1/E2 and IAV HA^34^; VEEV E, VSV, and EBV^35^. Unless stated otherwise, EBOV GP was used as the fusion protein and C15:1 GA was used to inhibit fusion. As shown in Fig. 2B, C15:1 GA inhibited fusion for the eight tested fusion proteins. Figure 2C documents that fusion was blockaded by GA C13:0, C15:1, or C17:1.

### Statistical analysis

For HCMV plaque assays, data were analyzed using one-way ANOVA analysis in SPSS. For BI6 n = 25; degrees of freedom between groups = 4 and within groups = 20. The F value is 68.827. For CH19 n = 24; degrees of freedom between groups = 4 and within groups = 19. The F value is 41.090.

For fusion experiments, Pair-wise Student t-tests were used to compare the outcome of a manipulation on fusion as compared to the control. Error bars are SEM (n ≥ 3).

HCMV DNA copy numbers expressed as percent of control. Significance was set at P < 0.05. P values were obtained using a two-tailed t-test. All measurements were performed in duplicate. Standard curves were included on each run.

For ZIKV, the data were analyzed using unpaired, two tailed student’s t test and represented as mean± standard error. Experiments were done independently at least three times and a value of p ≤ 0.05 indicated a statistically significant difference.

## Supplementary information

Supplementary Information.

## Data Availability

All data generated or analyzed during this study are included in this published article and its supplementary information files, or they have been placed in public repositories.
