# Author Correction: Ginkgolic acid inhibits fusion of enveloped viruses

**DOI:** 10.1038/s41598-020-64445-y

**Published:** 2020-05-05

**Authors:** Ronen Borenstein, Barbara A. Hanson, Ruben M. Markosyan, Elisa S. Gallo, Srinivas D. Narasipura, Maimoona Bhutta, Oren Shechter, Nell S. Lurain, Fredric S. Cohen, Lena Al-Harthi, Daniel A. Nicholson

**Affiliations:** 1 Department of Neurological Sciences, Rush University Medical Center, Chicago, IL USA; 2 Department of Microbiology and Molecular Cell Biology, Eastern Virginia Medical School, Norfolk, VA USA; 3 Department of Physiology and Biophysics, Rush University Medical Center, Chicago, IL USA; 4 Independent researcher, Chicago, IL USA; 5 Department of Microbial Pathogens and Immunity, Rush University Medical Center, Chicago, IL USA

Correction to: *Scientific Reports* 10.1038/s41598-020-61700-0, published online 16 March 2020

In the original version of this Article, Nell S. Lurain’s affiliation was incorrectly listed as ‘Independent researcher, Chicago, IL, USA’. The correct affiliation is listed below.

Department of Microbial Pathogens and Immunity, Rush University Medical Center, Chicago, IL, USA

This error has now been corrected in the PDF and HTML versions of the Article.

