# Supplementary material for: Ginkgolic acid inhibits fusion of enveloped viruses
Source: Sci Rep. 2020 Mar 16;10:4746. doi: 10.1038/s41598-020-61700-0 (PMC7075884; doi:10.1038/s41598-020-61700-0)
Supplement: Supplementary file 1 — Supplementary Information. [file 41598_2020_61700_MOESM1_ESM.pdf]

Supplementary Information

Ginkgolic acid inhibits fusion of enveloped viruses

Ronen Borenstein[1, 2*], Barbara A. Hanson[1], Ruben M. Markosyan[3], Elisa S. Gallo[4], Srinivas D. Narasipura[5], Maimoona Bhutta[2], Oren Shechter[2], Nell S. Lurain[5], Fredric S. Cohen[3], Lena Al-Harthi[5], Daniel A. Nicholson[1*]

1. Department of Neurological Sciences, Rush University Medical Center, Chicago, IL, USA.
2. Department of Microbiology and Molecular Cell Biology, Eastern Virginia Medical School Norfolk, VA, USA.
3. Department of Physiology and Biophysics, Rush University Medical Center, Chicago, IL, USA.
4. Independent researcher.
5. Department of Microbial Pathogens and Immunity, Rush University Medical Center, Chicago, IL, USA.

* Corresponding author: Ronen Borenstein, BorensR@evms.edu,
Daniel A Nicholson, daniel_nicholson@rush.edu

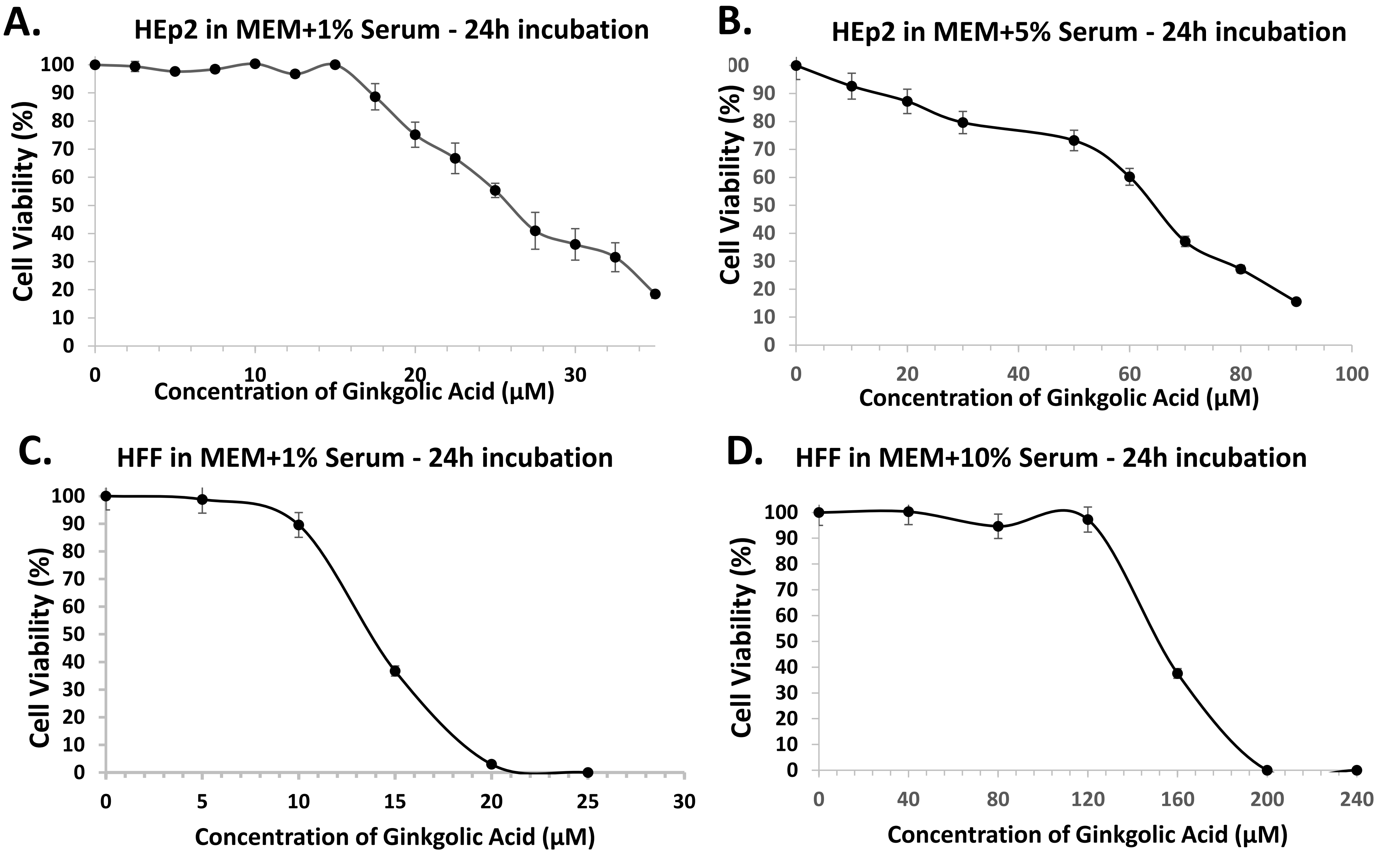

Figure S1. GA C15:1 cytotoxicity to HEp2 and HFF cells.

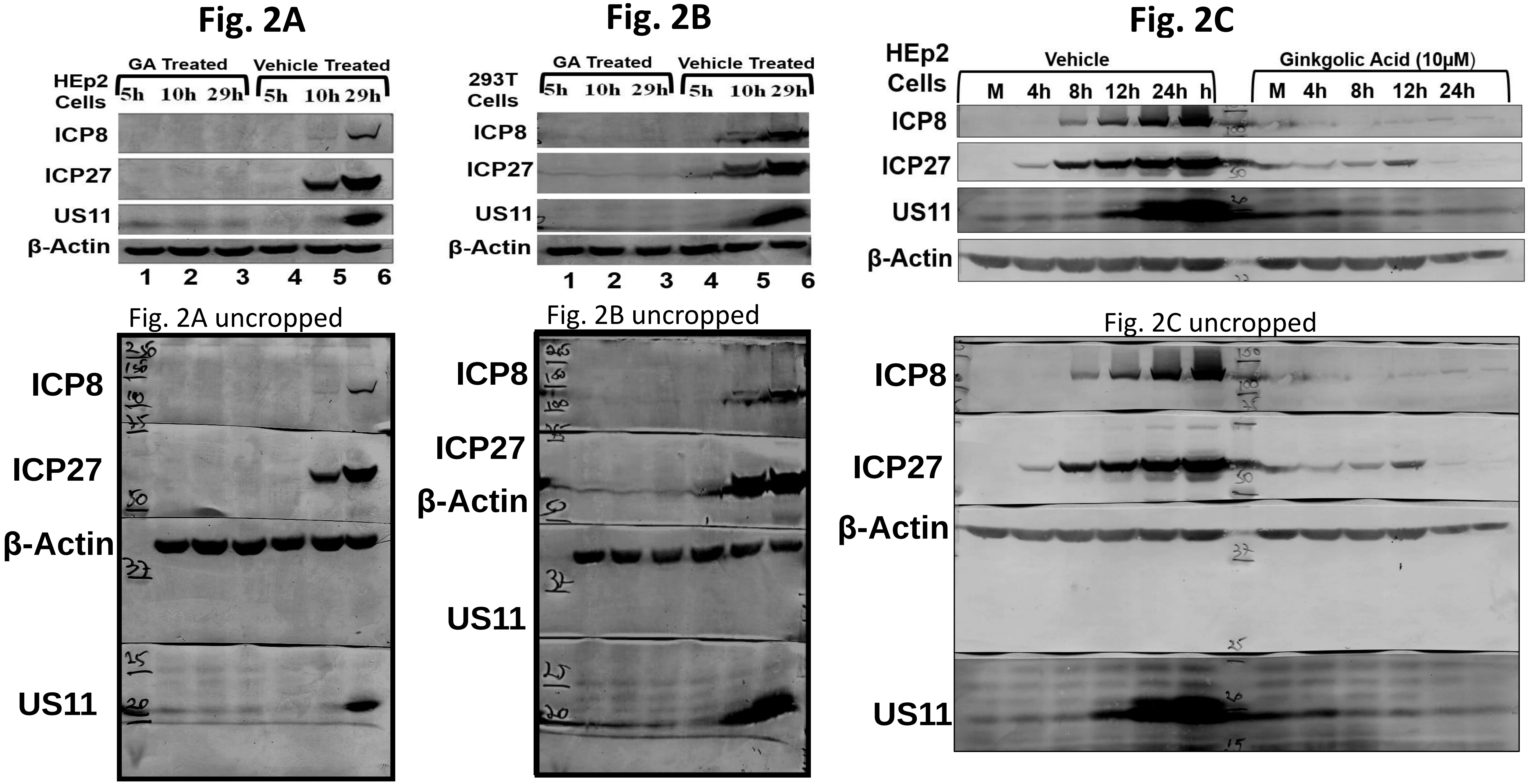

Figure S2. Fig 2 and the original uncropped images.

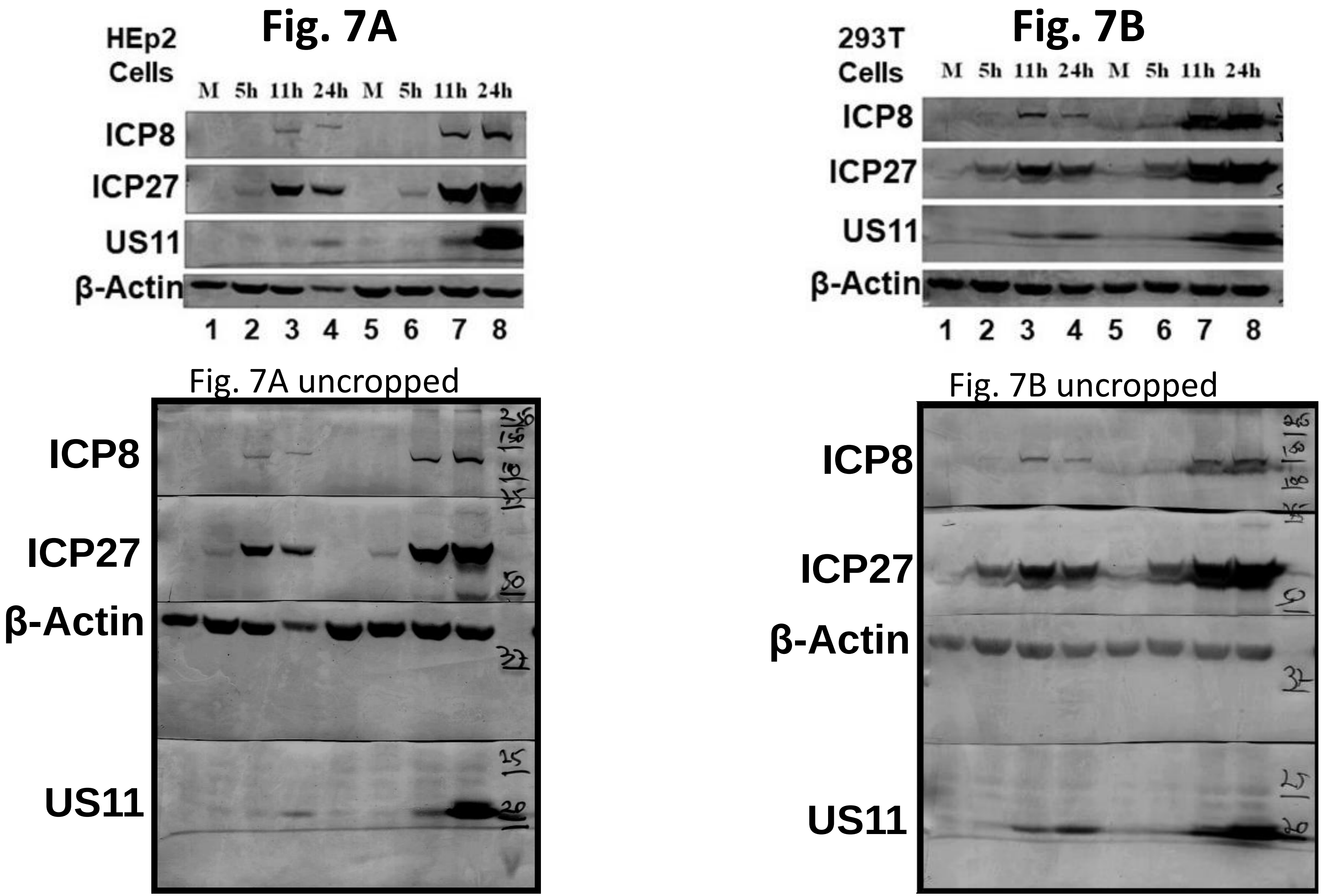

Figure S3. Fig 7 and the original uncropped images.

Table S1

Ginkgolic Acid	Structure
C15:1 $C_{22}H_{34}O_3$	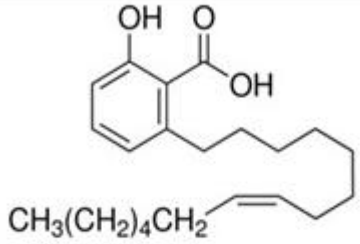
C17:1 $C_{24}H_{38}O_3$	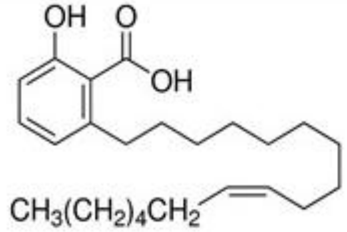
C13:0 $C_{20}H_{32}O_3$	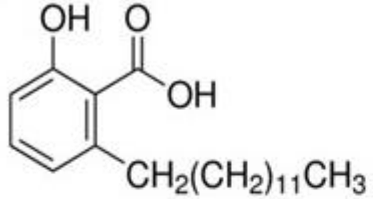

Table S2

	Virus	Fusion protein vector	Reference	Fusion protein class
1	Zika Virus (ZIKV)	pcDNA-ZIKV E-la	Shan-Lu Liu, Ohio State University	Class I
2	Human Immunodeficiency Virus (HIV)	Stable cell line TF228.1.16 constitutively expressing gp160 of the T-tropic BH10 strain of HIV-1	a gift from Dr. Z.L. Jonak (SmithKline Beecham, Philadelphia, PA). AIDS Res Hum Retroviruses. Jonak ZL et al, 1993 Jan;9(1):23-32., Melikyan GB et al, J Cell Biol. 2000 Oct 16;151(2):413-23.	
3	Ebola Virus (EBOV)	A mucin-deleted EBOV GP construct was obtained from David Sanders (Purdue University, West Lafayette, IN).	Markosyan et al, PLoS Pathog. 2016 Jan 5;12(1).	
4	Influenza A virus	The plasmid encoding IAV HA (Thailand KAN-1/2004 H5N1 strain), was provided by Gary Nabel (NIH, Bethesda, MD).	Li et al, PLoS Pathog. 2013 Jan;9(1).	
5	Semliki Forest Virus (SFV)	pCB3-wt vector. This vector, provided by Dr. Margaret Kielian, Albert Einstein College of Medicine, encodes the SFV structural proteins E1, p62 (precursor of E2), 6K, and capsid. Transfecting produces surface expression of the E1 fusion protein and the mature E2 protein.	Markosyan RM, Kielian M, Cohen FS. J Virol. 2007 Oct;81(20):11218-25.	Class II
6	Venezuelan Equine Encephalitis Virus (VEEV)	The plasmid was provided by Dr. Robert Davey, Texas Biomedical Research Institute, San Antonio, Texas.	Markosyan and Cohen (2013). PLoS One. 2013 Oct 4;8(10).	
7	Vesicular Stomatitis Virus (VSV)	The plasmid was obtained from Dr. Douglas Lyles (Wake Forest University).	Markosyan and Cohen (2013). PLoS One. 2013 Oct 4;8(10).	Class III
8	Epstein-Barr Virus (EBV)	The plasmid EBV-gB801was obtained from Dr. Richard Longnecker (Northwestern School of Medicine).	Markosyan and Cohen (2013). PLoS One. 2013 Oct 4;8(10).	
